# Vaccine Development in the Time of COVID-19: The Relevance of the Risklick AI to Assist in Risk Assessment and Optimize Performance

**DOI:** 10.3389/fdgth.2021.745674

**Published:** 2021-11-02

**Authors:** Quentin Haas, Nikolay Borisov, David Vicente Alvarez, Sohrab Ferdowsi, Leonhard von Meyenn, Douglas Teodoro, Poorya Amini

**Affiliations:** ^1^Risklick AG, Spin-off University of Bern, Bern, Switzerland; ^2^Clinical Trial Unit Bern, University of Bern, Bern, Switzerland; ^3^HES-SO University of Applied Sciences and Arts Western Switzerland, Geneva, Switzerland; ^4^Department of Radiology and Medical Informatics, University of Geneva, Geneva, Switzerland

**Keywords:** artificial intelligence, pharmacology, COVID-19, vaccine, risk analysis

## Abstract

The 2019 coronavirus (COVID-19) pandemic revealed the urgent need for the acceleration of vaccine development worldwide. Rapid vaccine development poses numerous risks for each category of vaccine technology. By using the Risklick artificial intelligence (AI), we estimated the risks associated with all types of COVID-19 vaccine during the early phase of vaccine development. We then performed a postmortem analysis of the probability and the impact matrix calculations by comparing the 2020 prognosis to the contemporary situation. We used the Risklick AI to evaluate the risks and their incidence associated with vaccine development in the early stage of the COVID-19 pandemic. Our analysis revealed the diversity of risks among vaccine technologies currently used by pharmaceutical companies providing vaccines. This analysis highlighted the current and future potential pitfalls connected to vaccine production during the COVID-19 pandemic. Hence, the Risklick AI appears as an essential tool in vaccine development for the treatment of COVID-19 in order to formally anticipate the risks, and increases the overall performance from the production to the distribution of the vaccines. The Risklick AI could, therefore, be extended to other fields of research and development and represent a novel opportunity in the calculation of production-associated risks.

## Introduction

The 2019 coronavirus (COVID-19) pandemic revealed an urgent need for the acceleration of vaccine development worldwide (1). Within months of the start of the COVID-19 pandemic, several pharmaceutical companies announced their objective to develop vaccines against COVID-19 by using different technologies (2–4). Each of the technologies presents advantages and pitfalls regarding the development of efficient COVID-19 vaccines (5–7). However, the situation requires that all the alternatives should be explored to shorten the course of the COVID-19 pandemic (8, 9). An increased speed of development induces risks, which must be taken seriously (10, 11). Hence, we analyzed the risks of the different technologies to produce the vaccines proposed by the pharmaceutical companies providing vaccines against COVID-19. This analysis was performed using scientific publications from early 2020 and utilizing the artificial intelligence (AI)-based search engine Risklick (12, 13). By calculating the risk score using a Probability and Impact Matrix and a semi-automated AI interface, we estimated the major risks faced by the pharmaceutical companies providing the vaccines.

A year later, our risk analysis on COVID-19 vaccine development was compared to the contemporary situation. Overall, we observed that the AI-based analysis anticipated the shortage of production and distribution faced by the pharmaceutical companies providing the RNA vaccines. The AI-based analysis highlighted the other issues faced by the pharmaceutical companies providing the vaccines, such as the delayed arrival of vaccines based on technologies other than RNA. Each issue faced by the vaccine providers is, as expected in the analysis, connected to the technology of their vaccine. Hence, our analysis exposes how AI-based technologies could become essential to the future of treatment development and the anticipation of associated risks.

## Methods

### Data

The Risklick AI collects and updates clinical trial data from a wide variety of sources such as the Clinical Trials Registr and datasets from the WHO each day. The metadata related to the publications are acquired from 14 international sources [BioRxiv; MedRxiv; Medline; Embase; Pubmed; Cinahl; Web of Science; Scopus; Cochrane; the International Clinical Trials Registry Platform (ICTRP); Dimensions; Living Evidence; Kaggle Cord-19 Dataset; and Google Scholar] allowing superior search performance relative to other acknowledged scientific publications search engines (12, 13). The Risklick AI is able to find and process COVID-19 references more effectively in terms of precision, F1 score, and recall, compared to the baseline platforms. Additionally, this living evidence management tool processes data in an automated manner, allowing analysis to be performed in a short amount of time. In total, 34 publications providing risk analysis on different vaccine technologies were gathered on June 10, 2020 (Supplementary Figure 1). These publications were used to evaluate the different risks in the development of the different vaccine technologies against COVID-19. The Risklick AI is available at www.risklick.ch.

### Selection of Variables

In this study, seven different types of vaccines were investigated: DNA and RNA based, inactivated virus, virus-like particle, live attenuated virus, protein subunit, and viral vector. The risks associated with vaccine production were then measured across seven categories: safety, cost of goods sold, manufacturing scalability, manufacturing process, shipping, and duration of immunity. Each category was defined in Supplementary Table 2.

### Risk Analysis

Using the set of publications, the risk for each vaccine technology for all the individual categories of risk was measured by using the Risklick semi-automated interface allowing the Probability and Impact Matrix calculation (14, 15). The probability of the risk happening (or “likelihood”) was categorized as “remote, unlikely, possible, likely, or certain” for each vaccine technology. These likelihood marks graded risk from lowest risk (remote) to highest risk (certain). Each of the grades was attributed a numeric value from one to five. Then, the potential impact of such a risk happening for each vaccine technology was categorized as “insignificant, minor, moderate, major, or critical.” These impact marks graded impact from lowest (insignificant) to highest (critical). Each of the grades was attributed a numeric value rising from one to five. The risk score of all the individual risk categories in each vaccine technology was calculated with the likelihood multiplied by the impact score. Visualization of risks per technology was performed by using radar charts, where all the risks were represented in the percentage of maximum risk per category based on the scores from Table 1. Risks were first analyzed on July 28, 2020, and compared with the situation a year later on May 22, 2021.

**Table 1:** Risk score of the different categories of risk for each vaccine technology.

	**DNA-based**	**RNA-based**	**Inactivated virus**	**Live attenuated virus**	**Protein subunit**	**Viral vector**	**VLP**
Safety	8	6	15	15	2	12	4
Cost of good sold	2	3	20	20	6	6	6
Manufacturing scalability	4	8	10	10	2	20	16
Manufacturing process	3	12	5	5	2	20	6
Time of availability	20	5	10	8	12	4	25
Shipping	1	10	2	3	4	9	4
Duration of immunity	25	20	1	2	20	12	12

### Validation

Verification and validation procedures were performed by two independent immunologists. All risk assessments, likelihood measurements, and impact estimations were analyzed and verified through non-automated input of the researchers.

## Results

**Figure 1:**
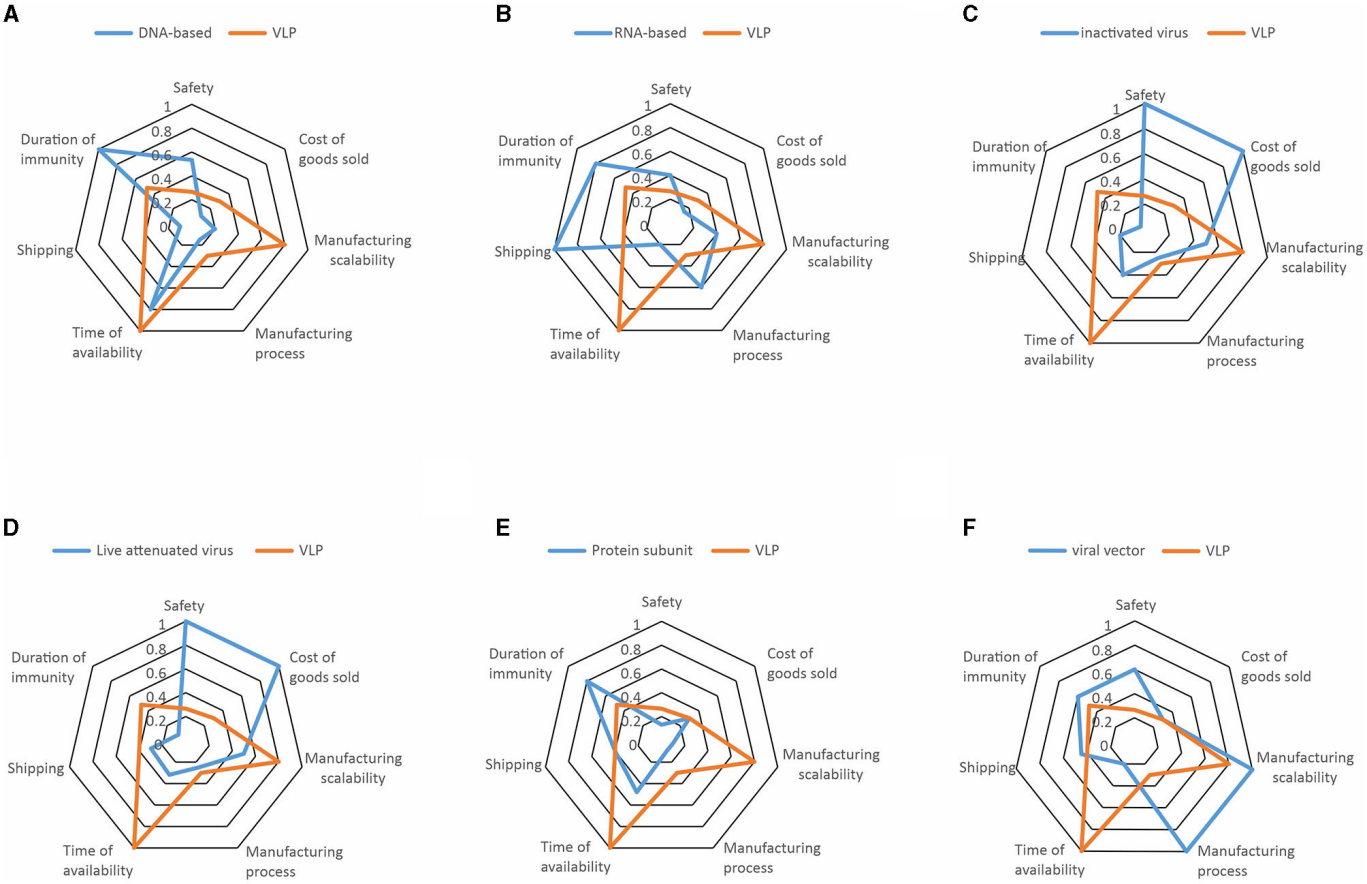
The radar charts of the seven different vaccine technologies illustrating the major risks estimated by risk score, expressed in percentage. Risks represented are safety, cost of goods sold, manufacturing scalability, manufacturing process, time of availability, shipping, and duration of immunity. Each technology is represented in blue while being compared to virus-like particle (VLP) in orange. Risk associated to **(A)** DNA-Based, **(B)** RNA-based, **(C)** inactivated virus, **(D)** live attenuated virus, **(E)** protein subunit, and **(F)** viral vector vaccines are presented. The most important risks point toward 100, while less important risks point toward 0.

The COVID-19 pandemic revealed the necessity for accelerated vaccine development worldwide (3, 7). In this critical context, we performed a risk analysis on the different vaccine technologies proposed by the manufacturers against COVID-19 in the early months of 2020 by using the AI-based search engine Risklick (12, 13). We graded seven different risks across seven different vaccine technologies by using a Probability and Impact Matrix (as illustrated in Supplementary Figure 1, for the risks linked to safety for each vaccine). Then, the risk score of every risk category for each vaccine technology was calculated. Risk calculation was limited by the low number of available publications at the time. An ideal quantitative risk assessment would require more data to reduce the limitations and strengthen the conclusions of the analysis. This resulted in a table of risks for each individual vaccine technology (Table 1). In this table, we already noticed that each vaccine technology possessed strengths and weaknesses different from one another, indicating the potential critical issues for the development of future vaccines. To simplify the visualization of the risks, a resume of risks per technology was performed using radar charts (Figure 1). To allow comparison, all technologies of the vaccines were compared to virus-like particle (VLP). This choice was made in the perspective of VLP being the least probable vaccine technology to effectively reach the market against COVID-19 in the near future. There are currently only four VLP-based COVID-19 vaccines in clinical trials and even in the optimistic cases, production could not reach more than 100 million doses before 2022 (16). This was confirmed in analysis with VLP presenting a particularly high risk for the time to availability and manufacturing scalability.

In June 2020, we already observed that the pharmaceutical companies providing vaccines would face different issues. Taken separately, DNA-based vaccines have the highest risk of providing only short-term immunity (Table 1, Figure 1). Shipping is a particular risk for the RNA-based vaccines and, to a lesser degree, viral vectors, which require extremely low temperatures. Inactivated viruses and live attenuated viruses raised concerns regarding their safety and the cost of goods sold. Protein subunits appear less risky across most categories, but the duration of immunity and time of availability still appeared as a considerable risk. Finally, the viral vectors presented serious risks connected to production, particularly for the categories of th manufacturing scalability and manufacturing process.

After 1 year, we compared our results to the contemporary situation (May 22, 2021). Presently, 18 different vaccines are approved in one or more countries (17). When selecting the vaccines with authorization in more than two countries, this number falls to 10 (three inactivated viruses, five virus vectors, and two RNA-based vectors).

First, we noticed that an extremely low number of the vaccines we classified as “high risky” managed to reach market authorization so far (these being inactivated virus, live attenuated virus, and VLP; Table 1). Moreover, based on the WHO database (18), the most widely represented technology in the clinical phase of the vaccine development is the protein subunit, which was also the least risky technology according to our analysis (Figure 1). However, none of the vaccines using this technology have reached market authorization to date. In fact, one of the major risks of the protein subunit compared to the competitors was its relatively important time to availability (Figure 1), which was much lower in the RNA-based vaccines and viral vectors. Hence, the major risk associated with the protein subunit vaccine might be the reason why vaccines based on such technology are slower to reach approval compared to vaccines based on the other technologies, despite the high number of clinical trials running using this technology at the time this article was written.

With respect to the other technologies, RNA-based vaccines were rapidly commercialized probably due to low cost and high scalability of production, which translate into an extremely low risk for the time to availability (Supplementary Figure 1). However, their high risk associated with shipping was verified when supply chains started suffering from storage temperature issues following market authorization (19). With respect to the viral vector technologies, the major risk was associated with manufacturing. This was demonstrated by the shortfall of the Oxford/AstraZeneca doses in Europe for 2021 (20) and Sputnik in Latin America (21). Hence, the major risk calculated in 2020 appeared to play a critical role in the deployment of the viral vector vaccines today as it did for the RNA vaccines. With respect to vaccine distribution, the early stage of vaccine distribution in 2021 highlighted difficulties with respect to the cold chain distribution process (22). This situation provoked the evident risks of supply chain failures and caused an important pressure on vaccine supply chain infrastructures (23, 24). Such risks will need to be addressed in the future, particularly from the perspective of mRNA vaccine distribution (25). Altogether, the risk analysis performed using the Risklick AI in early 2020 offered an accurate prediction of risks that would be faced by the different pharmaceutical companies providing vaccines in the year 2021.

## Discussion

The COVID-19 outbreak resulted in one of the biggest waves of publications in the history of modern science (26, 27). This influx of COVID-19-related data made literature retrieval and monitoring one of the greatest challenges of the pandemic (28). The COVID-19 pandemic highlighted the need for vaccine developers and health authorities to provide general guidance for the faster development and preparation of vaccines while offering the highest safety and efficacy standards (1, 29). Shortly after the start of the COVID-19 pandemic, several pharmaceutical companies providing vaccines announced their objective to develop vaccines against COVID-19, using different technologies, all within a matter of months (2, 3).

The choice of the type of vaccine is crucial: carrier or vector, adjuvant, excipients, dosage form, and route of administration; all directly impact the potential efficacy of the vaccine against COVID-19, but also the logistics of manufacturing, storing, and distributing the vaccine might affect the availability of the product (5, 6, 9). Hence, this simple variation in the composition of the vaccines will directly impact the policy of mass vaccination and the access to vaccine doses worldwide, affecting the duration of the COVID-19 pandemic (8).

In this context, we performed a risk analysis on the different technologies used by the pharmaceutical companies providing vaccines against COVID-19 in the early months of 2020 by using the AI-based search engine Risklick (12, 13).

Our results demonstrate that the AI-based analysis of risks allows the identification of the vaccine category with the highest chances of success. Moreover, the Risklick AI allowed us to foresee the different issues pharmaceutical companies providing vaccines would face, notably during the production and distribution phases of their product.

Our results highlight how AI-based technologies will become essential in the development of various therapies in the future. Moreover, our results demonstrate that, by using in-depth risk analysis, the global performance of vaccine developers could be increased by anticipating major issues, allowing corrective, and preventive actions. Such an approach, if generalized, would represent an important gain of time and efficiency for every scientist and manufacturer involved in providing care and drugs. The adoption of detailed risk analysis by all the scientists and manufacturers involved in the COVID-19 pandemic could represent a game-changer that could positively impact the future of therapy development worldwide.

## Data Availability Statement

The original contributions presented in the study are included in the article/Supplementary Material, further inquiries can be directed to the corresponding author/s.

## Author Contributions

PA designed the study. QH and NB wrote the manuscript. QH, PA, and LM performed the experimental work. DA, SF, NB, and DT developed the AI models. All authors had full access to the data, helped draft the report or critically revised the draft, contributed to data interpretation, reviewed, and approved the final version of the report.

## Funding

This study was funded by the Innosuisse project funding number 41013.1 IP-ICT.

## Conflict of Interest

QH, NB, LM, and PA are work for Risklick. The remaining authors declare that the research was conducted in the absence of any commercial or financial relationships that could be construed as a potential conflict of interest.

## Publisher's Note

All claims expressed in this article are solely those of the authors and do not necessarily represent those of their affiliated organizations, or those of the publisher, the editors and the reviewers. Any product that may be evaluated in this article, or claim that may be made by its manufacturer, is not guaranteed or endorsed by the publisher.

## Supplementary Material

Supplementary Figure 1 The Probability and Impact Matrix for the safety of the six different vaccine technologies.

Supplementary Table 1 List of publications used for the semi-automated artificial intelligence (AI) risk calculation.

Supplementary Table 2 Definition of the variables used to assess the risk of vaccine technologies.
