# Supplementary figures and images for: Vaccine Development in the Time of COVID-19: The Relevance of the Risklick AI to Assist in Risk Assessment and Optimize Performance
Source: Front Digit Health. 2021 Nov 2;3:745674. doi: 10.3389/fdgth.2021.745674 (PMC8593331; doi:10.3389/fdgth.2021.745674)

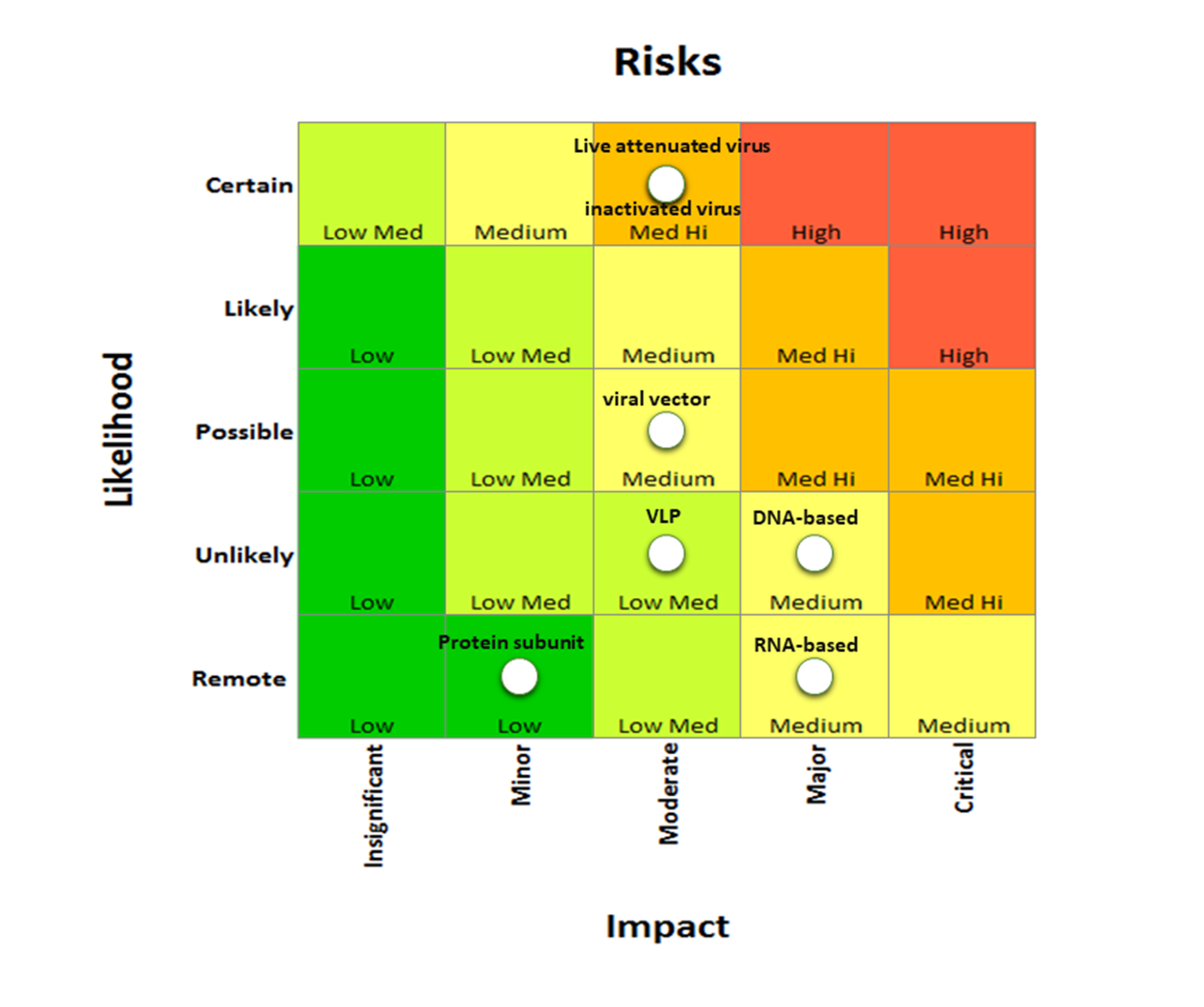

Supplement: Supplementary Figure 1 — The Probability and Impact Matrix for the safety of the six different vaccine technologies. [file Image_1.TIF]
